# Development and Validation of the ScAbIeS Tool for Diagnosing Scabies by Community Healthcare Workers in Resource-Limited Settings

**DOI:** 10.7759/cureus.42268

**Published:** 2023-07-21

**Authors:** Priyamadhaba Behera, Debkumar Pal, Chandra S Sirka, Binod K Patro, Dinesh P Sahu, Siddhartha Dash, Manish Taywade

**Affiliations:** 1 Community Medicine and Family Medicine, All India Institute of Medical Sciences, Bhubaneswar, Bhubaneswar, IND; 2 Dermatology, All India Institute of Medical Sciences, Bhubaneswar, Bhubaneswar, IND; 3 Dermatology and Venereology, All India Institute of Medical Sciences, Bhubaneswar, Bhubaneswar, IND; 4 Community and Family Medicine, All India Institute of Medical Sciences, Bhubaneswar, Bhubaneswar, IND

**Keywords:** diagnostic performance, screening tool, validation study, diagnosis of scabies, asha (accredited social health activist) workers

## Abstract

Introduction

Scabies can lead to community outbreaks if not diagnosed early. Developing and validating a relevant tool for diagnosing scabies at the community level is essential to bridging the early diagnosis and treatment gap.

Objective

The objective of this study is to develop and validate a newer tool to diagnose scabies at the community level by Community Healthcare Workers (CHWs) in resource-limited settings.

Methods

The developed "ScAbIeS" tool comprised five items divided into two major and three minor criteria. After its development, a longitudinal descriptive study validated the "ScAbIeS" tool. The eligible participants were included in the study through active screening in villages under the Rural Health Training Center (RHTC) Mendhasala. Those villages' Accredited Social Health Activists (ASHS) were included as CHWs for diagnosing scabies using the "ScAbIeS" tool. The participants with skin lesions and/or itching were diagnosed with scabies by CHWs using the "ScAbIeS" tool. The diagnosis of CHWs using the "ScAbIeS" tool was compared with those of physicians, including expert dermatologists, to determine the sensitivity and specificity.

Results

Kappa’s agreement is found to be 0.896 for CHWs and trained physicians regarding the diagnosis of scabies by the "ScAbIeS" tool. Cronbach’s alpha is 0.738 for major criteria and 0.565 for minor criteria. 0.778 is found to be Cronbach’s alpha for the total scale. The "ScAbIeS" tool is 85% sensitive and 100% specific to diagnose scabies when used by CHWs.

Conclusion

The "ScAbIeS" tool can be used to diagnose scabies at the community level by CHWs with appropriate training. It will lead to the prevention of complications and community outbreaks of scabies.

## Introduction

A microscopic ectoparasite known as Sarcoptes scabiei var. hominis is the cause of scabies, a skin condition [1]. At any point in time, more than 200 million people are suffering from scabies worldwide [2]. The global age-standardized Disease Adjusted Life Years (DALYs) per 100,000 people attributed to scabies were 62.53 (95% CI 34.67-99.87) for both sexes [3]. Scabies usually manifests as intense itching at night with skin lesions. The signs consistent with scabies are the presence of burrows, erythematous papules, nodules, and vesicles, alongside pruritus or evidence of excoriation [4]. Though scabies is seen mainly in children, it is not uncommon in adults. In cases of delayed treatment, there can be complications like persistent itching, insomnia, secondary bacterial infections, Norwegian scabies, and disease outbreaks in the community [5]. Scabies can even cause life-threatening complications like glomerulonephritis and rheumatic heart disease due to secondary infection by Streptococcus spp. [6,7].

Physicians mostly diagnose Scabies based on clinical features and history. The presence of erythematous papular lesions in typical body parts like the axilla, web of fingers of hands and feet, groin, and inside of wrists, also known as the cycle of Hebra, is commonly associated with a diagnosis of scabies [8,9]. The similar features in other family members also strongly suggest a scabies infection [10]. In very few cases, mites can be seen by non-invasive higher-power imaging devices or light microscopy of potassium hydroxide (KOH) mounts from a skin lesion [11]. The diagnosis of scabies with only symptoms or a family history of skin lesions is neither sensitive nor specific [12].

The World Health Organization (WHO) enlisted scabies as a neglected tropical disease in 2017. There is a persistent gap in the treatment of scabies in resource-limited settings due to a lack of trained physicians and dermatologists [13]. Being a contagious disease, a delay in treatment can lead to outbreaks in the community and complications [5,10]. In India, a large community-level health workforce exists under the National Health Mission (NHM). Accredited Social Health Activists (ASHA), Auxiliary Nurse Midwifery (ANM), and Anganwadi Workers (AWW) have been working as Community Healthcare Workers (CHWs) for many years in the improvement of maternal and child health, along with providing other health-related services [14]. In most places, ANM gets training in midwifery or nursing, and ASHA and AWW are educated formally until class eight. They live close to the community and have an advantage in the cultural milieu. Early detection and management of scabies are essential to limiting community outbreaks in resource-limited settings. Therefore, CHWs may play a vital role in screening and linking scabies cases to Health and Wellness Centers (HWCs) and Primary Health Centers (PHCs). This study aimed to develop and validate a tool for diagnosing scabies in CHWs.

## Materials and methods

Tool development

The research team (PB, CS, DP, and DPS) primarily conducted a desk review regarding the signs and symptoms of scabies. Four items, divided equally into major and minor criteria, were identified after consensus among the research team members. Five items were finalized after interaction with ASHAs during the training; two were major and three were minor (Table 1). Two of the major and one of the three minor criteria should be present for diagnosing scabies at the community level. Major criteria consist of the presence of erythematous papular lesions and the distribution of those lesions in a typical anatomical position. Minor criteria include lesion density (in typical anatomical sites), itching more during the night, and similar clinal features in family contacts.

**Table 1:** Domains and items of the "ScAbIeS" tool

Key domains of tool	Items
Major criteria	Skin lesion (morphology of lesions: erythematous cutaneous papular lesion)
	Anatomical distribution of skin lesions (lesions over finger web-space, axilla, medial side of palm and hand, groin, genitalia, breast)
Minor criteria	Itching (preferably at night)
	Similar symptoms or lesions among contacts (symptoms-itching, signs-skin lesions)
	Density of papules more in the finger web-space, axilla, medial side of palm and hand, groin, genitalia, and breast than in other sites of the body

Tool validation

**Figure 1:**
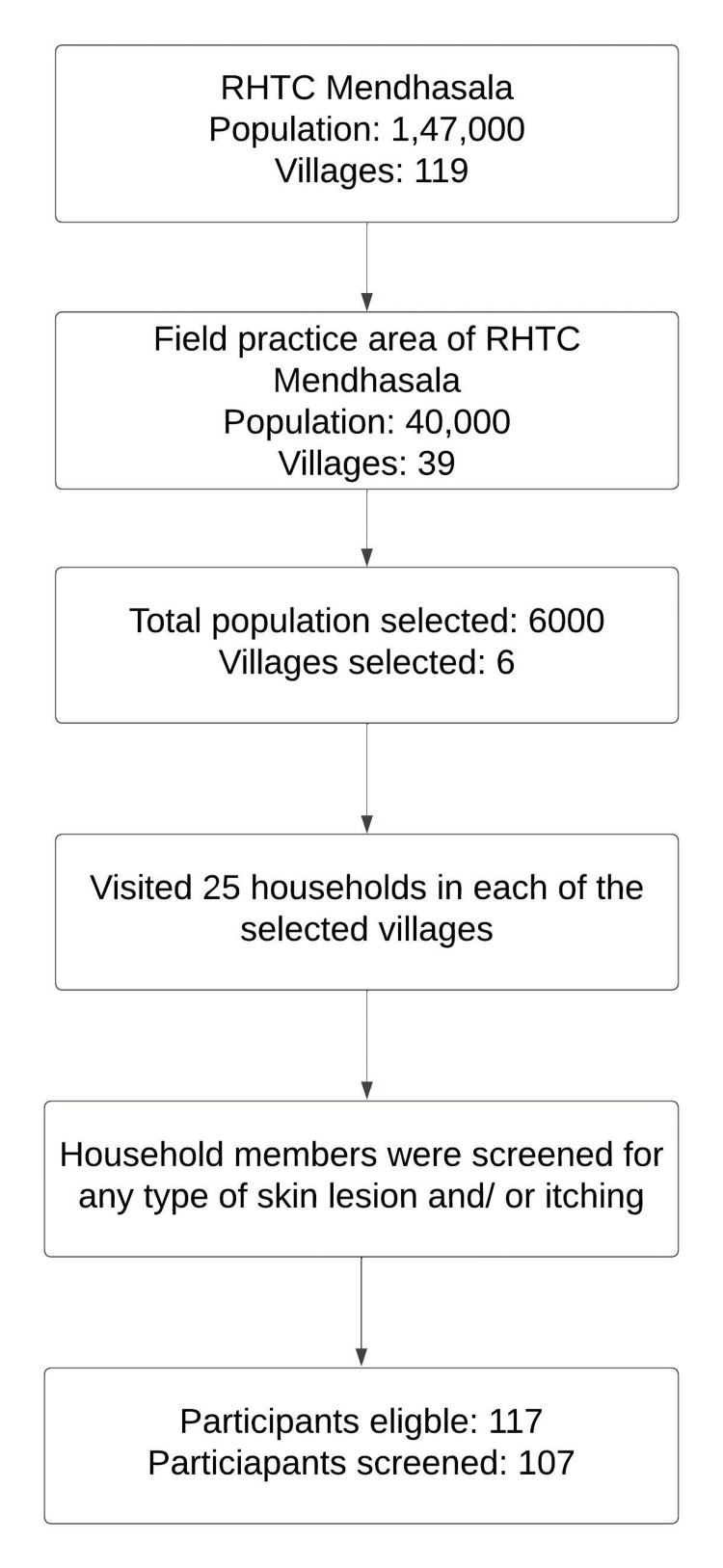
Flow diagram showing procedure of selection of study participants

We conducted this longitudinal descriptive study in the rural field practice area of the Department of Community Medicine and Family Medicine of the All India Institute of Medical Sciences, Bhubaneswar (AIIMS, Bhubaneswar). AIIMS Bhubaneswar is a tertiary care and teaching institute. Its rural field practice area is situated in the rural Bhubaneswar block of the Khordha district of Odisha, a state in eastern India. All the people residing in the field practice area who have skin lesions and/or itching are included in this study, irrespective of age and sex. The total population of the study area is around 1.5 lakh, distributed into 119 villages. Six of the 119 villages were selected, with a population of around 6,000 (Figure 1). Simultaneously, six ASHAs were selected who are working in those villages. Two expert dermatologists (CS and SD) trained ASHAs before the initiation of the study using a scabies case scenario. Pre-test and post-test questionnaires were used to assess the effectiveness of the training provided by ASHA. The study was implemented after getting at least 90% correct responses in the post-test assessment from all the ASHAs. One researcher (DP) visited those villages door-to-door with ASHA workers to screen and include their participation in the study. ASHAs screened the patients using the tool, and physicians confirmed the diagnoses using clinical expertise, which was further verified by two expert dermatologists (CS and SD).

Sample size estimation and statistical analysis

With an assumed 10% prevalence of scabies in the study area and an expected 90% agreement between ASHA and trained physicians in diagnosing scabies cases, the minimum sample size required was 107 to achieve a minimum power of 80% with an alpha error of 5% [2]. With a 10% exclusion rate, the final sample size was estimated at 119. The data are entered in Microsoft Excel (Microsoft® Corp., Redmond, WA), and descriptive analysis is done using it. The quantitative data were presented with a mean and standard deviation (SD), whereas the categorical data were presented with a proportion and 95% confidence interval (CI). Cronbach’s alpha was calculated for internal reliability, and Cohen’s kappa was calculated for agreement among ASHAs and physicians. Statistical Package for Social Sciences version 26 (IBM SPSS, Chicago, IL, USA, software version 26.0) was used for calculating Cronbach’s alpha. Sensitivity and specificity are calculated for the tool using clinical diagnosis by a physician as the gold standard.

Ethical issues

We obtained ethical approval from the Institutional Ethics Committee (IEC) of AIIMS Bhubaneswar before the initiation of the study (IEC Number: T/IM-NF/CM&FM/21/171). ASHA workers were included in the study after receiving written informed consent from them. The information was provided to all of the participants in their local language. Participants aged more than 18 years were included in the study after giving written informed consent. Participants aged 12-18 years were included after receiving written informed consent from them along with written informed consent from one of their parents/legal guardians. Participants aged 7-11 years were included after receiving verbal informed consent from them along with written informed consent from one of their parents/legal guardians. Participants aged less than five years were included after receiving written informed consent from one of the parents/legal guardians of the participants. This method of consent was approved by the IEC of AIIMS Bhubaneswar.

## Results

Scabies was diagnosed when two of the major and one of the three minor criteria were present for any participant after the consensus of all experts. We included 107 participants who fulfilled the inclusion criteria after approaching 117 eligible participants. One hundred and seven recruited participants were distributed among six villages, where five ASHAs were involved in the study. The mean age of participants was 20.17 years (SD 13.17 years). There was a significant difference in age between the two groups of scabies (7±2.8 years) and non-scabies (23.2±14.5). The agreement was found to be 89.6% among ASHAs and trained physicians for diagnosing scabies in participants. All of the diagnoses were confirmed by trained physicians. Cronbach’s alpha was calculated as 0.738 and 0.565, respectively, for major and minor criteria, whereas the alpha value is 0.778 for the tool as a whole. The tool was 85% (95% C.I.; 62.11-96.79%) sensitive and 100% (95% C.I.; 95.85-100.00%) specific compared to the physician’s clinical diagnosis (Table 2).

**Table 2:** Sensitivity and specificity of "ScAbIeS" tool (diagnosis of scabies of ASHA vs. physician) (n=107)

	Scabies detected by a physician N (%)	Non-scabies detected by a physician N (%)
Scabies detected by ASHA	17 (15.89%)	00 (00.00%)
Non-scabies detected by ASHA	03 (02.80%)	87 (81.31%)

The distribution of items among all participants is mentioned in the table (Table 3).

**Table 3:** Distribution of participants with respect to different items of "ScAbIeS" tool (n=107)

Items	Erythematous papular lesion, N (%)	Lesion at the anatomical site N (%)	Nocturnal itching, N (%)	Difference in density of lesion, N (%)	Family contact, N (%)
Scabies (N=17)	17 (100.00%)	17 (100.00%)	17 (100.00%)	13 (76.47%)	12 (70.50%)
Non-scabies (N=90)	25 (27.78%)	00 (0.00%)	54 (60.00%)	00 (0.00%)	3 (3.33%)

## Discussion

The "ScAbIeS" tool was developed through desk review and consensus among physicians and expert dermatologists. The dermatologists also confirmed the validity of the tool with their expertise. The scabies tool is found to be 85% sensitive when used by ASHA workers after training. The higher sensitivity proved the criterion validity of the tool. The high agreement between ASHA and physicians confirmed the utility of this tool at the community level.

The mean age of scabies patients is similar to the study conducted by Nair et al. [13]. In this study, the most typical symptom associated with scabies was found to be an erythematous papule, similar to earlier studies [8,10]. Earlier, the International Alliance for Control of Scabies (IACS) tool was available for physicians to classify different types of scabies [12]. The IACS tool defines confirmed scabies as the presence of mites/eggs/feces on light microscopy from the skin, the detection of mites/eggs/feces in a high-powered imaging device, or the visualization of mites using dermoscopy. Clinical scabies is defined as the presence of either burrows or typical lesions in the male genitalia, or the presence of a typical distribution of typical lesions with itching and contact with scabies cases. Suspected scabies is defined as typical lesions in a typical distribution with either itching or contact with scabies cases. Suspected scabies also includes atypical lesions or atypical distribution with itching and contact with scabies patients. Many items in IACS cannot be used at the community level due to feasibility. However, a few of the items of the IACS, like typical lesions in typical distribution with itching, are similar to the "ScAbIeS" tool.

As the ASHAs can use this tool, we can assume that this tool can be used by teachers, ANMs, and AWWs after training, as the ASHA workers' intellect level is assumed to be lower than that of AWWs, ANMs, and school teachers. Teachers, AWWs, and ANMs come into contact with many children during routine activities, leading to an effective community-based strategy for early diagnosis of scabies and early initiation of treatment. This strategy is very crucial in pockets in areas with indigenous people and difficult terrain, specifically where the burden of scabies is higher in comparison to other areas [9]. The high sensitivity of this tool will enable it to diagnose scabies at the community level and link scabies patients to a primary health care center for early treatment initiation.

Limitation

We did not encounter any Norwegian scabies; the ability of this tool is limited for detecting those types of scabies patients. Norwegian scabies is a rare phenomenon that is usually not encountered during daily clinical practices. It can be found only in severely immunocompromised, debilitated, or elderly patients. The education level of ASHAs included in our study was higher than class eight; however, based on the selection criteria of the ASHAs, they may have a lower education. Also, the level of motivation and training among ASHAs should be kept in mind when implementing this strategy at the country level. The wide confidence interval of sensitivity can be attributed to the smaller sample size of the study. Further studies with a larger sample size will estimate the sensitivity of the "ScAbIeS" tool with better precision.

## Conclusions

The agreement between the diagnosis of ASHA using the "ScAbIeS" tool and the physician's diagnosis was 89.6%. The "ScAbIeS" tool has high sensitivity (85%) and specificity (100%) at the community level when used by CHWs. The "ScAbIeS" tool has two major criteria and three minor criteria. For the identification of scabies, the requirements that have to be fulfilled are two major criteria and one or more minor criteria. The tool can be used to diagnose scabies at the community level by CHWs with appropriate training. This tool will help CHWs detect scabies early through linkage to the health care system. This has the potential to constitute part of an effective community-based strategy for scabies control/elimination.

## Appendices

Steps for the training of ASHAs

Step 1: Pre-training assessment of ASHA workers regarding their knowledge of scabies using a questionnaire in the local language.

Step 2: Images of skin lesions related to scabies were shown to ASHA workers.

Step 3. ASHA workers were oriented to the items and domains of the "ScAbIeS" tool.

Step 4: A practical demonstration with five scabies cases, including mild, moderate, and severe cases, and ten non-scabies cases with similar presentations (itching and/or skin lesions) was done.

Step 5: Post-training assessment of ASHA workers regarding their knowledge of scabies using a questionnaire in the local language.
